# Genetic Variability, Character Association, and Path Analysis for Economic Traits in Menthofuran Rich Half-Sib Seed Progeny of *Mentha piperita* L.

**DOI:** 10.1155/2014/150830

**Published:** 2014-05-22

**Authors:** Birendra Kumar, Himanshi Mali, Ekta Gupta

**Affiliations:** Seed Quality Lab, Genetics and Plant Breeding Division, CSIR-Central Institute of Medicinal and Aromatic Plants (CIMAP), P.O. CIMAP, Lucknow 226015, India

## Abstract

Menthofuran rich eight half-sib seed progeny of *Mentha piperita* (MPS-36) were studied for various genetic parameters, namely, coefficient of variation, heritability, genetic advance, correlation, and path of various plant and oil attributes, namely, plant height, L : S ratio, herb yield, **β**-myrcene, limonene, 1,8-cineole, menthone, menthofuran, neomenthone, pulegone, and menthol. Maximum genotypic coefficient of variation and genetic advance as percentage of mean were recorded for pulegone, followed by menthofuran and 1,8-cineole. The genotypic correlation in general was higher than phenotypic; positive significant correlation was recorded for limonene with 1,8-cineole and menthone, **β**-myrcene with limonene, and 1,8-cineole and menthofuran with neomenthol. A high direct positive effect on menthofuran was of neomenthol.

## 1. Introduction

Peppermint (*Mentha piperita *L.), an aromatic herb that arose as a sterile (hexaploid) hybrid between spearmint (*Mentha spicata*) and water mint (*Mentha aquatica*) over 250 years ago [1, 2] belonging to family Lamiaceae, is distributed widely in temperate and sub-temperate climatic regions. The fresh herb on distillation yields essential oil containing a large variety of aroma chemicals in varying composition. Peppermint leaves and their oil possesses medicinal properties as carminative, stimulant and used for food and flavouring purpose. Menthofuran possessing a characteristic note is an important marker phytomolecule of peppermint oil. Although there are some other natural sources of menthofuran apart from* M. piperita*, like* M. aquatica* (aqua mint; having a limited distribution), most of the industrial demand for the molecule is met through synthetic menthofuran (derived from isopulegol) [3]. Maximal menthofuran content in* M. piperita* has been reported to be 25% in the oil [4]. An estimated demand of menthofuran in aroma industry is 150–200 mt/year [5]. In view of the high cost of synthetic menthofuran, a breeding program was carried out to develop menthofuran rich genotypes through half-sib seed progeny. Although* Mentha piperita* has now couple of high yielding varieties [6], they have not been used so far in study of correlation and path analysis with the result that no* a priori* knowledge is available on the selection parameters for oil yield and quality [7, 8]. It was, therefore, essential to study the characters associations and path analysis for both the dependent and independent variables: oil yield and menthofuran content potential (menthofuran is the major quality constituent) in peppermint. The present study was planned with menthofuran rich genotypes to have an idea of interrelationships among the economic traits for developing a suitable selection strategy in menthofuran rich half-sib seed progeny of peppermint.

## 2. Materials and Methods

The material comprised menthofuran rich peppermint genotype MPS-36 (developed as open pollinated seed progeny of variety Kukrail in 2000) and its eight half-sib seed progeny of* Mentha piperita* with identity as MPS-36 (1), MPS-36 (2), MPS-36 (3), MPS-36 (4), MPS-36 (5), MPS-36 (9), MPS-36 (12), and MPS-36 (15). The genotype MPS-36 is characterized by presence of high menthofuran content with low menthol content and having flowering habit with ability of producing viable seeds. The experiment was conducted in the four consecutive years successfully to begin with the year of 2010 at CSIR-CIMAP experimental farm, Lucknow (India). The planted runners were done in the experimental plot in the fashion of row randomized block design with three replications in the month of January every year and standard agronomic practices were followed to raise a healthy and representative crop of* Mentha piperita*.

Samples were collected from 50 cm at middle length of line avoiding border effect on growth of crop on 115 days after planting. Essential oil was distilled from all the samples taking 200 g of fresh herb weight basis. Composite sample of herb was used for oil content estimation through Clevenger type apparatus. Observations on the quality traits, namely, *α*-pinene, *β*-pinene, *β*-myrcene, limonene, 1,8-cineole, menthone, menthofuran, isomenthone, pulegone, and menthol, were recorded through gas liquid chromatography on CP-3800 Varian Gas Chromatograph using SUPELCOWAX 10 capillary column (30 M × 0.32 mM × 0.25 *μ*M). The oven temperature was programmed from 40−120 @ of 3°C/min initial hold of 9 min; then raised 120–140°C @ of 2°C/min with hold 2 min; then again raised 140–220°C @ of 5°C/min with final hold of 2 min; injector and detector temperature were 250°C. Hydrogen is used as a carrier gas with flow of 1.7 ml/M and split 1 : 40. The data were proceeding on star chromatography data system. Peak identification is based on retention time of component.

The observations on morphometric traits, namely, plant height, L : S ratio, herb yield, and oil content (%), were recorded in the month of April every year (2010–2013) at the maturity of crop. The mean data were subjected to correlation and path coefficient analysis (partial regression approach) following the methods described by Dewey and Lu [9]. The statistical analysis for variance using statistical software SPAR-1 of IASRI, New Delhi, was available in the Department of Genetics and Plant Breeding CSIR-CIMAP Lucknow. Variability for different qualitative character was estimated. Heritability and genetic advance were calculated following standard procedures.

## 3. Results and Discussions

The ANOVA has revealed that the progeny are highly significant for all quantitative traits except *β*-myrcene, which was significant at 0.05% level. Among quantitative traits plant height and herb yield are highly significant except L : S ratio and oil content, which was non-significant (Table 1). The magnitudes of range for quantitative as well as qualitative characters were wide indicating the possibilities of exploiting the available variability for further genetic improvement programmes. The magnitudes of phenotypic coefficients of variations were invariably higher than genotypic coefficient of variations indicating the influence of environment for quantitative traits, while for qualitative traits, phenotypic and genotypic coefficients of variations were the same indicating strict genetic control on these traits (Table 2). Estimates of heritability and genetic advance were higher for quantitative traits barring L : S ratio and oil content, where genetic advance was quite low indicating that this trait may be under influence of nonadditive genetic control. In respect to quantitative traits the poor estimates of heritability and genetic advance indicate that inheritance of these traits is being influenced by interallelic interaction rather than intra-allelic interaction. Knowledge of intercharacter relationship is very important in plant breeding for indirect selection of the characters that are not easily measured (oil content and oil quality) and for those that exhibit low heritability. However, under a complex situation the estimates of correlation alone may be often misleading due to mutual cancellation of component traits, so it becomes necessary to study the path coefficient analysis simultaneously which takes in to account the casual relationship in addition to the degree of relationship [10, 11].

In general, genotypic correlation was higher than corresponding phenotypic correlation for most of the character pairs (Table 3), which could be due to modifying effect of environment on association of characters at the genetic level [12–14]. The genotypic and phenotypic correlation coefficient among 12 traits revealed that highly positive significant correlation was recorded for limonene with 1,8-cineole and menthone, highly negative significant genotypic correlation was recorded for plant height with L : S ratio (Table 3). Further, to have clear understanding the genotypic association of oil quality traits with menthofuran where partition into their direct and indirect effect through path coefficient analysis was displayed in Table 4. The path analysis is a statistical technique used primarily to examine the comparative strength of direct and indirect relationship among variables and thus permits a critical examination of components that influence a given correlation and can be helpful in formulating an efficient selection strategy [15, 16]. The path coefficient analysis of* Mentha piperita* for menthofuran content are given in Table 4. The limonene had maximum positive direct effect on menthofuran content (0.591) followed by neomenthone (0.557). On the other hand menthol has maximum direct negative effect (−0.793) followed by 1,8-cineole (−0.543), pulegone (−0.427), and *β*-myrcene (−0.279) on menthofuran content. The *β*-myrcene had negative direct effect (−0.279) on and negative genotypic correlation with menthofuran and also showed negative indirect effect via 1,8-cineole, menthone, and neomenthone. The limonene had direct positive effect (0.591) but showed maximum negative indirect effect via 1,8-cineole (−0.460) for menthofuran content. The neomenthone had direct positive effect (0.557) as well maximum positive genotypic correlation for menthofuran content and showed positive indirect effect via other quality traits except limonene (−0.263) and pulegone (−0.215). It showed the improvement in neomenthone content will also lead to enhancement in menthofuran content simultaneously. However, the other quality traits had negative correlation and negative direct effect indicates that care should be taken to consider these traits in selection strategies for further improvement of menthofuran content.

**Figure 1:**
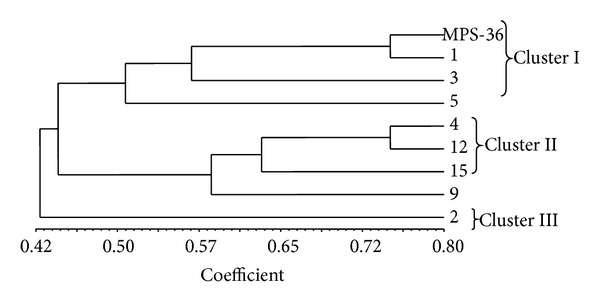
Dendrogram showing phylogenetic relationship among nine accessions of* Mentha piperita *L. (MPS-36) based on morphological traits.

The morphological data of eight genotypes along with parent were used to construct phenogram for understanding the phylogenetic relationship among them (Figure 1). The phenogram classified nine accessions of* Mentha piperita* into three major clusters, namely, clusters I, II, and III. Clusters I and II each contained four genotypes; however cluster III is unique and had only one genotype, that is, MPS-36 (2). The parent (MPS-36) was grouped in cluster I along with genotypes MPS-36 (1), MPS-36 (3), and MPS-36 (5), which indicates that these genotypes are closer to parent with respect to morphological traits studied. However, the other four genotypes, that is, MPS-36 (4), MPS-36 (12), MPS-36 (15), and MPS-36 (9), were grouped in cluster II showing little morphological variation with respect to parental line. It was interesting to note that the genotype MPS-36 (2) showed quite different morphological variation and formed a single cluster.

**Figure 2:**
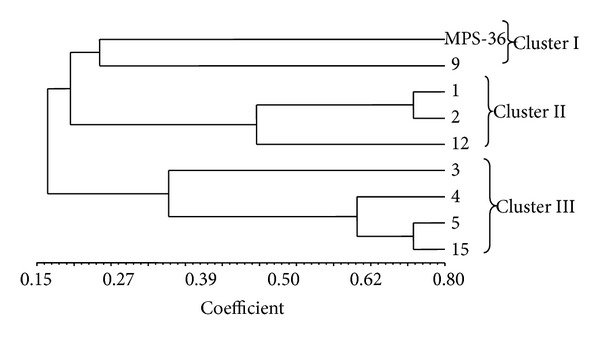
Dendrogram showing clustering of genotypes based on chemoprofiling of* Mentha piperita* L. (MPS-36).

To understand the phylogenetic relationship, the clustering was also performed based on chemical profiling of all the genotypes used in the present investigation (Figure 2). Here, all the genotypes were also grouped in three major clusters with different compositions of genotypes. Cluster I had parent (MPS-36) and genotype MPS-36 (9). The second cluster had three genotypes, that is, MPS-36 (1), MPS-36 (2), and MPS-36 (12). Cluster III had four genotypes, that is, MPS-36 (3), MPS-36 (4), MPS-36 (5), and MPS-36 (15). It was noticed that there was no congruence between morphological and chemoprofiling based clustering. The genotype which was very unique in morphological clustering, that is, MPS-36 (2), was clustered with different genotypes in chemoprofiling clustering. There were only two genotypes, that is, MPS-36 (4) and MPS-36 (15), which were grouped together in the same cluster.

An attempt was made in the present study to explore the possibility of augmenting the productivity and quality in peppermint by deployment of half-sib seed progeny selection breeding approach. Kumar et al. [17] and Patra and Kumar [18] amply demonstrated in peppermint (*M. piperita*) and menthol mint (*M. arvensis*), respectively, that remarkable genetic variability which remains latent in the existing vegetatively propagated heterozygous clones of the popular varieties (Kukrail and Shivalik varieties of the two species, resp.) can be made available for breeding work by raising their open pollinated seed progeny. As reported by these authors, the oil content range in the OPSPs of* M. arvensis* cv. Shivalik varied between 0.37 and 1.08. In all the breeding programmes, irrespective of the crops yield improvement being the primary objective, judicious selection is practiced for higher yields. GCV, broad sense heritability and genetic advanced for the yield and quality attributes was displayed in Table 2. GCV has been as high as 120.13%; such genetic variation offers an opportunity for better means of selection and augmentation of pulegone in nine half-sib seed progeny. Though genotypic coefficient of variation measures the amount of variation in character, it is not possible to access the amount of heritable variation based on this estimate. Burton and de Vane [19] have suggested that GCV along with heritability estimates would provide a better idea of amount of genetic gain expected through phenotypic selection. High heritability coupled with high genetic advance observed for herb yield was under additive genetic control and simple selection for these traits would be quite effective. Broad sense heritability estimates are expected to be high because total genetic variance on which these estimates are based is made up of three parts, namely, additive genetic variance, nonadditive genetic variance due to dominance, and nonadditive genetic variance due to nonallelic gene interactions.

## 4. Conclusion

Considered together, the correlation and results led for predicting high menthofuran content in indirect selection, the neomenthone would be reliable major parameter for selection of elite half-sib genotype having desirable menthofuran content. As demonstrated by the result of the present study, a potent breeding technique like selection in half-sib seed progeny (the technique which also has been used in developing the superior variety Kosi) has been able to ensure improvement in oil quality beyond 50%.

## Figures and Tables

**Table 1:** Analysis of variances (MSS) of 12 traits in 9 half-sib seed progeny of *Mentha piperita*.

Source	df	MSS											
		Plant Height	L : S Ratio	Oil Content	Herb Yield	*Β*-Myrcene	Limonene	1,8 Cineole	Menthone	Mentho-furan	Neo-menthone	Pulegone	Menthol
Replication	2	307.51	0.00034	0.00008	225.0	0.00006	0.00003	0.00002	0.00006	0.00024	0.00005	0.00024	0.00000
Treatment	8	235.70**	0.01482	0.00472	45677.0**	3.23042*	6.78816**	23.8014**	14.11664**	385.993**	14.4560**	688.044**	1740.32**
Error	16	44.23	0.00089	0.00104	1418.7	0.00003	0.00004	0.00004	0.00004	0.00002	0.00003	0.00009	0.00006

*P < 0.05, **P < 0.01.

**Table 2:** Estimates of genetic parameter in 9 half-sib seed progeny of *Mentha piperita*.

Characters	Range	Mean ± SE	Phenotypic coefficient of variation	Genotypic coefficient of variation	Heritability (h^2^b)	Genetic advance
Plant height	51.13–82.33	65.296 ± 5.430	15.92	12.25	59.1	12.65
L : S ratio	0.80–1.03	0.982 ± 0.024	7.52	6.94	84.0	0.13
Oil content	0.23–0.35	0.309 ± 0.026	15.40	11.34	54.2	0.05
Herb yield	350.0–625.0	477.778 ± 30.754	26.62	25.42	91.2	50.02
*β*-Myrcene	0.42–2.96	1.347 ± 0.004	77.01	77.01	10.0	2.14
Limonene	2.84–6.55	4.778 ± 0.005	31.48	31.48	10.0	3.10
1,8 Cineole	0.41–6.79	3.277 ± 0.005	85.95	85.95	10.0	5.80
Menthone	3.51–10.19	6.864 ± 0.005	31.60	31.60	10.0	4.47
Menthofuran	0.37–36.20	11.799 ± 0.005	96.13	96.13	10.0	23.37
Neomenthone	1.48–7.60	3.593 ± 0.005	61.09	61.09	10.0	4.52
Pulegone	0.35–45.23	12.607 ± 0.008	120.13	120.13	10.0	31.20
Menthol	11.78–77.99	39.043 ± 0.006	61.69	61.69	10.0	49.62

**Table 3:** Genotypic (G) and phenotypic (P) correlation among 12 traits in 9 half sib seed progeny of *Mentha piperita*.

Characters		L : S ratio	Oil content	Herb yield	*β*-Myrcene	Limonene	1,8 Cineole	Menthone	Menthofuran	Neomenthone	Pulegone	Menthol
Plant height	P	−0.610	0.170	−0.056	0.163	0.104	−0.070	0.353	−0.361	−0.560	−0.269	0.150
	G	−0.870**	0.232	−0.088	0.212	0.135	−0.092	0.459	−0.469	−0.730*	−0.350	0.195

L : S ratio	P	—	0.184	0.161	−0.361	−0.286	−0.208	−0.501	0.082	0.392	0.158	0.288
	G	—	0.017	0.198	−0.394	−0.312	−0.227	−0.547	0.090	0.427	0.172	0.315

Oil content	P		—	−0.292	−0.458	−0.717*	−0.597	−0.575	−0.051	0.226	0.135	0.293
	G		—	−0.390	−0.622	−0.974**	−0.810**	−0.780*	−0.069	0.307	0.183	0.398

Herb yield	P			—	0.403	0.236	0.167	−0.079	0.101	0.089	−0.255	0.029
	G			—	0.423	0.246	0.175	−0.082	0.106	0.093	−0.267	0.031

*β*-Myrcene	P				—	0.689*	0.754*	0.476	−0.306	−0.302	−0.378	−0.098
	G				—	0.689*	0.754*	0.476	−0.306	−0.302	−0.378	−0.098

Limonene	P					—	0.848**	0.840**	−0.110	−0.444	−0.236	−0.336
	G					—	0.848**	0.840**	−0.110	−0.444	−0.236	−0.336

1,8 Cineole	P						—	0.678*	−0.221	−0.286	−0.403	−0.194
	G						—	0.678*	−0.221	−0.286	−0.403	−0.194

Menthone	P							—	−0.339	−0.718*	−0.453	−0.089
	G							—	−0.339	−0.718*	−0.453	−0.089

Menthofuran	P								—	0.788*	0.651	−0.789*
	G								—	0.788*	0.651	−0.789*

Neomenthone	P									—	0.503	−0.411
	G									—	0.503	−0.411

Pulegone	P										—	0.658
	G										—	0.658

*P < 0.05, **P < 0.01.

**Table 4:** Path coefficient analysis of *Mentha piperita* of menthofuran content.

Characters	*β*-Myrcene	Limonene	1,8 Cineole	Menthone	Neomenthone	Pulegone	Menthol	Genotypic correlation with menthofuran
*β*-Myrcene	**−0.279**	0.407	−0.409	−0.095	−0.168	0.161	0.078	−0.306
Limonene	−0.192	**0.591**	−0.460	−0.167	−0.248	0.101	0.266	−0.110
1,8 Cineole	−0.211	0.501	**−0.543**	−0.135	−0.159	0.172	0.154	−0.221
Menthone	−0.133	0.496	−0.368	**−0.199**	−0.400	0.193	0.071	−0.339
Neomenthone	0.085	−0.263	0.155	0.143	**0.557**	−0.215	0.326	0.788
Pulegone	0.106	−0.139	0.219	0.090	0.280	**−0.427**	0.522	0.651
Menthol	0.027	−0.198	0.105	0.018	−0.229	0.281	**−0.793**	−0.789

Residual = 0.0051.
